# Prostate Sarcoma: An Uncommon Diagnosis With Major Clinical Implications

**DOI:** 10.7759/cureus.86066

**Published:** 2025-06-15

**Authors:** Víctor M Molgado-Garza, Pedro A Madero-Morales, Andrés Guillén-Lozoya, Eliud López-Sánchez, Jesús R Garza-Hernández, Carlos Pacheco-Molina

**Affiliations:** 1 Urology, Hospital Universitario Dr. José Eleuterio González, Monterrey, MEX; 2 Urology, Mayo Clinic, Rochester, USA; 3 General Surgery, Hospital Universitario Dr. José Eleuterio González, Monterrey, MEX

**Keywords:** ileal conduit, leiomyosarcoma, luts, management, pelvic exenteration, prostate, sarcoma

## Abstract

Prostate sarcomas are rare and aggressive malignancies. It commonly presents with obstructive urinary symptoms and a normal prostate-specific antigen (PSA), making early diagnosis difficult due to its clinical overlap with benign prostatic conditions. These tumors tend to present at an advanced stage due to unspecific symptoms and their aggressive progression. Radical surgery remains the gold-standard treatment, but its impact on the overall prognosis remains limited, particularly in patients over 50 years of age with metastatic or locally advanced disease. We present the case of a 71-year-old male who presented with refractory severe lower urinary tract symptoms (LUTS) despite a previous transurethral resection of the prostate (TURP) and dual therapy. Imaging revealed an enlarged prostate (300 g) and repeated TURP procedures failed to relieve symptoms or identify malignancy until the histopathology from a subsequent procedure revealed prostate leiomyosarcoma. Further imaging demonstrated local extension to the bladder and rectum. A multidisciplinary team proceeded with total pelvic exenteration, including resection of the prostate, bladder, and rectum, and creation of an ileal conduit and colostomy. Postoperative pathology was positive for a T4 leiomyosarcoma, infiltrating bladder and rectum, with preserved seminal vesicles. The patient initially recovered but later developed hypovolemic shock from arterial bleeding and required additional surgeries. Despite intensive care and surgical efforts, he died on postoperative day 28 due to disseminated intravascular coagulation. This case highlights the diagnostic challenges, aggressive progression, and scarcity of treatment options for this condition, emphasizing the importance of early diagnosis and multidisciplinary management.

## Introduction

Primary prostatic sarcomas are rare malignant tumors of mesenchymal origin, accounting for less than 1% of all prostate cancers [1]. They usually exhibit an aggressive clinical course. It typically presents with non-specific symptoms and normal prostate-specific antigen (PSA) levels, making early diagnosis particularly challenging. The most common histological form in adults is leiomyosarcoma, and rhabdomyosarcoma is the most common in children [2]. Most of these patients present with high-grade lower urinary tract symptoms (LUTS) due to the large tumor size at presentation. Most are staged at a locally advanced or metastatic stage, usually contiguous organs such as the bladder and rectum. The gold standard treatment is surgical resection, with pelvic exenteration reserved for locally advanced disease [2]. Nevertheless, the prognosis is poor, especially in males >50 years old with positive margins or distant metastasis [1]. Scarce literature underscores the need for further research and standardized treatment guidelines.

## Case presentation

A 71-year-old Mexican male presented with severe lower urinary tract symptoms (LUTS). His history included type 2 diabetes, treated with metformin, and hypertension managed with losartan. Six years earlier, he underwent a transurethral resection of the prostate (TURP) that showed benign prostatic hyperplasia. Despite the procedure and ongoing treatment with tamsulosin and dutasteride, his symptoms persisted. PSA was low at 0.09 ng/dL, and ultrasound showed a grossly enlarged prostate, measuring 300 grams.

The urology team proceeded with a urethrocystoscopy followed by a staged TURP. He was discharged with clear urine. Histology confirmed benign tissue. A week after catheter removal, the patient developed gross hematuria, severe anemia (Hb: 6 g/dL), and urinary retention due to clot obstruction. A second cystoscopy and TURP were performed, revealing necrotic tissue and active arterial bleeding. Tissue samples taken then led to a diagnosis of prostate sarcoma.

**Figure 1:**
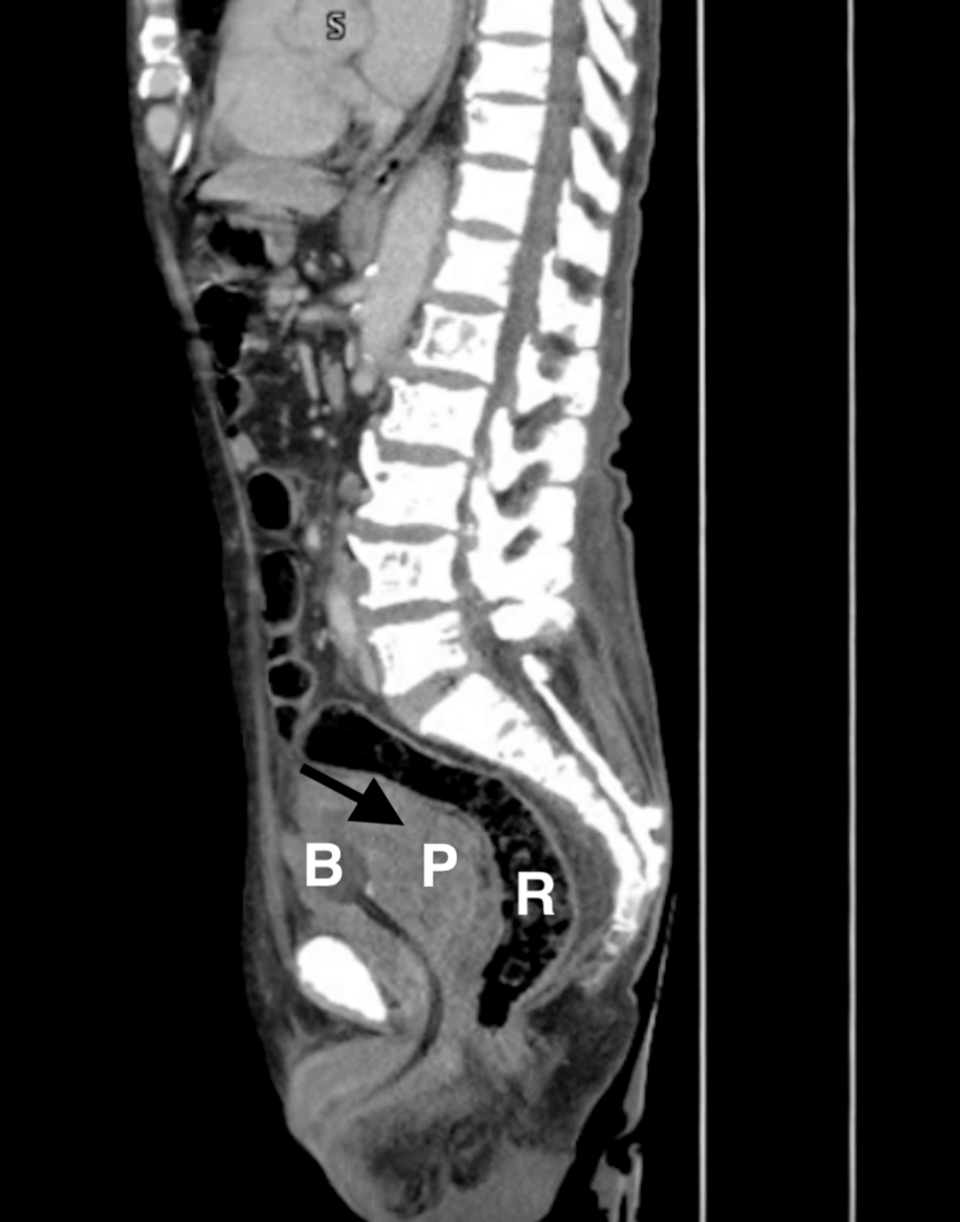
Contrast-enhanced abdominopelvic computed tomography (CT) in the sagittal plane demonstrates a markedly enlarged and heterogeneous prostate gland (arrow). There is loss of the normal fat planes between the prostate (P), bladder (B), and rectum (R), indicating direct tumor invasion into adjacent structures.

**Figure 2:**
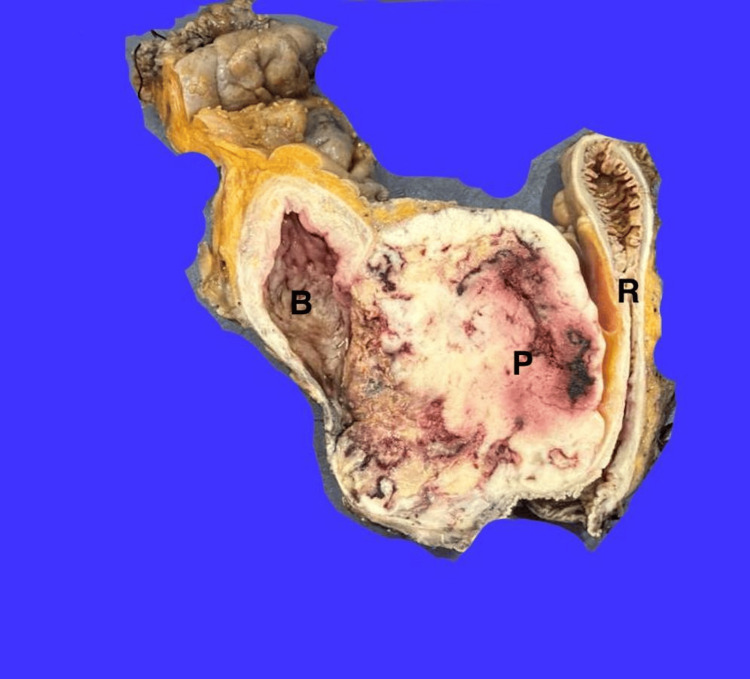
Macroscopic appearance of bladder (B), prostate (P), and rectum (R) from surgery.

A contrast-enhanced CT scan showed a large mass invading the bladder and rectum (Figure 1). A multidisciplinary team, including medical, urologic, and surgical oncologists, recommended total pelvic exenteration with an ileal conduit and colostomy. Surgery confirmed a large tumor compressing the sigmoid colon and encasing the rectum (Figure 2). The operation lasted seven hours with 500 cc blood loss. Postoperatively, his course was mostly smooth, apart from wound dehiscence on day three, which required revision. He was discharged on day seven.

**Figure 3:**
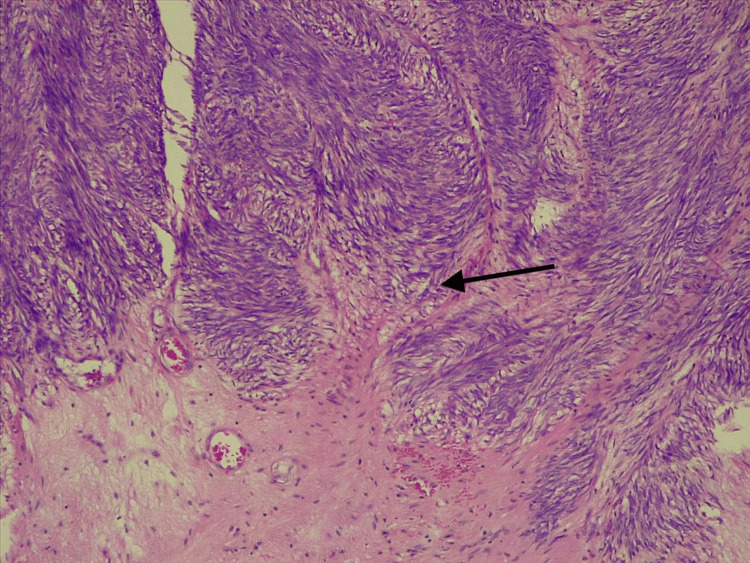
Microscopic pathology. H&E staining at 100x: this figure shows a prostatic stroma (arrow) neoplasm with tumor cells arranged in fascicles and necrosis.

**Figure 4:**
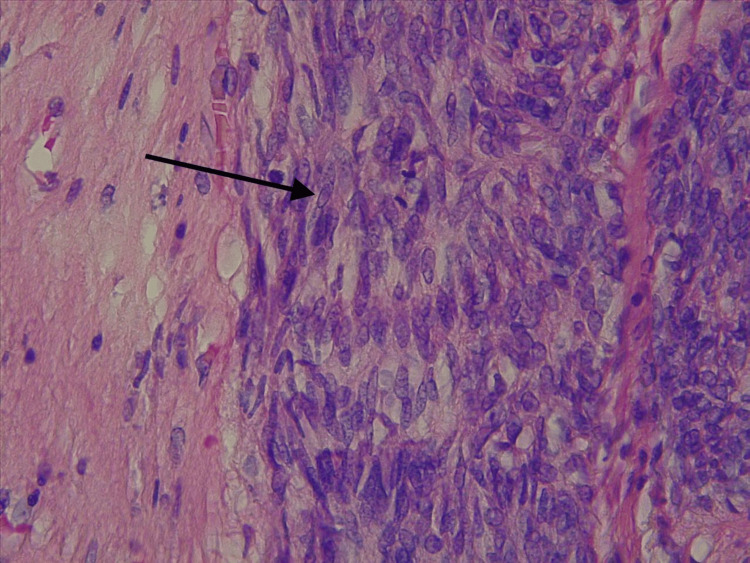
A 400x H&E staining field shows spindle cells with enlarged nuclei, coarse chromatin and marked pleomorphism (arrow), consistent with a prostatic leiomyosarcoma.

Histology revealed a pseudo-encapsulated, muscle-like tumor invading the bladder trigone and rectum, sparing the seminal vesicles (Figure 3). Microscopy revealed a spindle cell malignant neoplasm with pleomorphism, coarse chromatin, and necrosis, consistent with prostatic leiomyosarcoma (Figure 4).

On day 12, he returned with altered mental status and hypovolemic shock, unresponsive to fluids. Emergency surgery found bleeding from the left internal iliac artery, which was controlled. He was stabilized in the ICU and reoperated three days later for re-anastomosis of the ileal conduit and nephrostomy placement. Two days afterward, he developed recurrent bleeding from drains and ostomies. Labs showed disseminated intravascular coagulation. He died on postoperative day 28.

## Discussion

Primary sarcomas are malignant tumors that derive from the mesenchymal tissue of the ectoderm [1]. Prostate sarcomas are extremely rare tumors, usually presenting with a large obstructive mass and normal levels of prostate-specific antigen (PSA), with an aggressive outcome and poor prognosis, so early recognition and surgical treatment with curative intent offer patients the best chance of survival [3]. It is believed that approximately 50% of patients will die of their disease in the next two years [4]. It originates from prostatic stroma and, less commonly, from glandular elements. These tumors account for 0.1-0.7% of all primary prostate malignancies, so their rarity makes them difficult to diagnose and treat because the only available literature comes from a small series of case reports. The risk factors for recurrence and progression that have been identified, such as tumor grade, size, depth of invasion, and surgical margin status, are quite different in prostate sarcomas [4]. Musser et al. reported the largest cohort of adult prostate sarcoma cases worldwide, with 38 reported from 1982 to 2012 [4].

They are divided into the following subtypes in descending order: leiomyosarcoma, rhabdomyosarcoma, fibrosarcoma, spindle cell sarcoma, and a variant of the proliferation of stromal tumor of unknown malignant potential, or STUMP when they do not meet criteria for sarcoma [2]. Wang et al. described leiomyosarcoma as the predominant subtype of primary prostate sarcomas (40%), whereas rhabdomyosarcoma is more common in the pediatric population [2]. Previous reports suggest that rhabdomyosarcoma may be a favorable subtype, while others have reported no difference between subtypes [4]. The median age of diagnosis reported by Wang et al. is 37; nevertheless, patients diagnosed before age 50 have a better overall survival than those older than 50 years (OS 27 vs. 15 months) [2]. Cheville et al. described a cohort of 23 patients with the diagnosis of leiomyosarcoma. They found a mean age of diagnosis of 61, which may explain why these patients have the worst outcomes. One of the patients had prostate adenocarcinoma 10 years before the diagnosis of leiomyosarcoma and was treated with external beam radiation [5]. It has been reported that 72.6% of patients present with locally advanced disease and 24-45% have metastatic disease at presentation, possibly due to the lack of specific clinical symptoms, resulting in more advanced disease at the time of diagnosis [3,4].

Patients usually present with lower urinary tract symptoms, more commonly as urinary obstruction, because tumors can be quite large at the time of diagnosis, with a median of 9.5 cm. Wang et al. described dysuria as the most common presenting symptom in their series, with 72% [2]. Hematuria (24%), perineal pain, ejaculatory pain, constipation, and weight loss are reported. Only 21% of patients present initially with urinary retention [4]. Digital rectal examination (DRE) usually reveals an enlarged prostate in almost all patients [4]. They usually do not elevate PSA because these tumors do not originate from the epithelial tissue. The median serum PSA reported by Wang et al. was 1.39 ng/ml, with a range between 0.4 and 2.4 ng/ml described by Sexton et al. [2,6]. Thus, it can be suspected when a young patient presents with marked lower urinary tract symptoms, an enlarged prostate at DRE, and a normal PSA value. Almost all patients have locally advanced diseases with the bladder (most common), seminal vesicles, and rectal wall as the principal sites of involvement. The most common site of metastasis is the lung (17.6%) [2].

The initial diagnosis is made by image-guided needle biopsy; however, it is not rare that these tumors are diagnosed after transurethral resection of the prostate or other interventions for obstructive pathology of the prostate. Electron microscopy and immunohistochemistry are required to confirm the mesenchymal origin of these tumors and determine the subtype of prostate sarcoma [2]. Immunoreactivity can be seen for vimentin (100%) and actin (63%) [7]. One study revealed that 5% of patients with adenocarcinoma can have a sarcomatoid component [8]. Staging evaluation follows the same process as for other prostatic tumors, with CT, MRI, or PET-CT. MRI offers a better delineation of soft tissue planes and a superior assessment of the relationship between the tumor and adjacent viscera, facilitating surgical planning. Presentation at an age older than 50 years, leiomyosarcoma subtype, metastatic disease at the time of presentation, and lack of surgery with curative intent are risk factors for unfavorable outcomes [2]. Sexton et al. reported that negative surgical margins and the absence of metastasis at diagnosis are associated with a better prognosis, as these patients showed better overall survival. They demonstrated that patients with negative surgical margins have a five-year overall survival of 67% versus 0% for those with positive surgical margins [6,9]. Even with curative intent, the majority of patients in all the cohorts have died of the disease, with less than 5% disease-free [10]. Russo et al. found in their study that low-grade, small, and localized tumors were favorable predictors of long-term survival [7]. Musser et al. demonstrated in their series that all patients with positive surgical margins experienced recurrence and died due to disease progression. Primary treatment depends on whether or not metastatic disease was present at the time of diagnosis. This cohort reported that 55% of patients had no evidence of metastatic disease at the time of diagnosis. Of those with localized disease, 90% underwent extirpative surgery: 42% had cystoprostatectomy, 28% had radical prostatectomy, and 19% underwent pelvic exenteration [4].

Surgery has become the mainstay of treatment and usually involves cystoprostatectomy or total pelvic exenteration for locally advanced disease. Radical prostatectomy is reserved for those patients presenting with a small sarcoma confined to the prostate, but this presentation is extremely rare [2]. Outcomes in adults contrast with rhabdomyosarcomas in children, where the prognosis is generally better and chemotherapy plays an established role [1]. Neoadjuvant chemotherapy could have a role in patients with localized disease and downsize the tumor to facilitate surgical resection. Nevertheless, preoperative treatment with platinum-based agents and doxorubicin caused >95% necrosis of the tumor but with limited radiographic response [9]. Currently, no studies have specifically evaluated adjuvant chemotherapy; however, data from a large meta-analysis of soft tissue sarcomas have shown improved progression-free survival without a significant impact on overall survival [11]. Not surprisingly, the most important prognostic factor is the presence of metastatic disease at the time of diagnosis, underscoring the limitations of current systemic therapy for sarcoma in adults [3]. The protocols that are used include vincristine, dactinomycin, cyclophosphamide (VAC), or mesna, adriamycin, ifosfamide, and dacarbazine (MAID), although no durable response was seen with any systemic therapy [1]. This information contrasts with the pediatric population, where the majority of children with confined disease and nearly half of the patients with advanced disease are alive five years after initial diagnosis [12]. Novel targets such as imatinib, used for gastrointestinal stromal tumors, may be a reasonable alternative treatment for prostate sarcoma in the future [13].

In general, patients with prostate sarcomas have worse outcomes compared to those with extremity or soft tissue sarcomas as a result of multiple factors such as tumor biology, advanced stage at presentation, and, most importantly, the difficulty in achieving complete resection [7,9]. Regarding prognosis, Musser et al. reported a median cancer-specific survival of 2.9 years, with 7.7 years for localized disease and 1.5 years for metastatic disease, not quite different from the cohort of Wang et al., who reported a median overall survival of 23 months. Recurrence-free survival is 20 months, dropping to 10 months after metastasis [4,6]. Overall, the first, second, third, and fifth-year survival rates were 80%, 47.4%, 22.6%, and 11.3%, respectively [4].

There is no consensus on how the follow-up should be conducted for these patients; however, guidelines for soft tissue sarcomas provide us with insight into this topic. Office visits with history and physical examination every three months for the first three years, every six months for the next two years, and then annually, performing imaging studies with CT, MRI, or PET-CT every 3-6 months for three years, and then every 6-12 months thereafter. Again, this data is based on soft tissue sarcomas, but with the aggressive behavior of this neoplasm, closer surveillance must be warranted for detecting early recurrence [14].

## Conclusions

Prostate sarcomas are very aggressive neoplasms that clinicians should suspect in patients with severe lower urinary tract symptoms and marked enlargement of the prostate on digital rectal examination; however, these manifestations are nonspecific. There is no consensus to date on the optimal approach for managing these patients. Despite its rarity, it is important to make efforts to continue reporting these cases to establish a guide for clinicians in the management of prostate sarcomas.
